# Challenges in Determining the Role of Microbiome Evolution in Barrett’s Esophagus and Progression to Esophageal Adenocarcinoma

**DOI:** 10.3390/microorganisms9102003

**Published:** 2021-09-22

**Authors:** Caitlin Guccione, Rena Yadlapati, Shailja Shah, Rob Knight, Kit Curtius

**Affiliations:** 1 Division of Biomedical Informatics, Department of Medicine, University of California San Diego, La Jolla, CA 92093, USA; cguccion@ucsd.edu; 2 Bioinformatics and Systems Biology Program, University of California San Diego, La Jolla, CA 92093, USA; robknight@ucsd.edu; 3 Department of Pediatrics, University of California San Diego, La Jolla, CA 92093, USA; 4 Division of Gastroenterology, Department of Medicine, University of California San Diego, La Jolla, CA 92093, USA; ryadlapati@health.ucsd.edu (R.Y.); s6shah@health.ucsd.edu (S.S.); 5 Veterans Affairs, San Diego Healthcare System, San Diego, CA 92161, USA; 6 Department of Bioengineering, University of California San Diego, La Jolla, CA 92093, USA; 7 Center for Microbiome Innovation, University of California San Diego, La Jolla, CA 92093, USA; 8 Department of Computer Science and Engineering, University of California San Diego, La Jolla, CA 92093, USA

**Keywords:** esophageal adenocarcinoma, Barrett’s esophagus, *Helicobacter pylori*, microbiome evolution, esophagus microbiome

## Abstract

Esophageal adenocarcinoma (EAC) claims the lives of half of patients within the first year of diagnosis, and its incidence has rapidly increased since the 1970s despite extensive research into etiological factors. The changes in the microbiome within the distal esophagus in modern populations may help explain the growth in cases that other common EAC risk factors together cannot fully explain. The precursor to EAC is Barrett’s esophagus (BE), a metaplasia adapted to a reflux-mediated microenvironment that can be challenging to diagnose in patients who do not undergo endoscopic screening. Non-invasive procedures to detect microbial communities in saliva, oral swabs and brushings from the distal esophagus allow us to characterize taxonomic differences in bacterial population abundances within patients with BE versus controls, and may provide an alternative means of BE detection. Unique microbial communities have been identified across healthy esophagus, BE, and various stages of progression to EAC, but studies determining dynamic changes in these communities, including migration from proximal stomach and oral cavity niches, and their potential causal role in cancer formation are lacking. *Helicobacter pylori* is negatively associated with EAC, and the absence of this species has been implicated in the evolution of chromosomal instability, a main driver of EAC, but joint analyses of microbiome and host genomes are needed. Acknowledging technical challenges, future studies on the prediction of microbial dynamics and evolution within BE and the progression to EAC will require larger esophageal microbiome datasets, improved bioinformatics pipelines, and specialized mathematical models for analysis.

## 1. Introduction

Esophageal adenocarcinoma (EAC), a cancer that occurs in the distal esophagus often near the gastroesophageal junction, continues to be a major cause of cancer morbidity and mortality in the United States (US). Since 1975, there has been an estimated 5-fold increase in EAC incidence in the US [1]. Recent data trends suggest these rates have plateaued post-2010 [2]; nevertheless, screening and surveillance programs, and diagnostic and therapeutic advances, have yet to translate into meaningful declines in EAC incidence and mortality. The survival outcome for patients diagnosed with EAC is highly correlated with stage at diagnosis, with 5-year survival rates of 51.1%, 26.5% and 5% for patients at localized, regional and distant stages, respectively [2]. Unfortunately, of the approximately 10,000 new cases annually [3], 39.1% present with distant stage disease at diagnosis [2], with nearly half (~45%) of all patients dying within the first year of EAC diagnosis [2]. The alarming increase in EAC cases, along with its continued poor prognosis, despite diagnostic and therapeutic advances in this space, signals a knowledge gap in our understanding of EAC pathogenesis and risk of progression to EAC from preneoplastic tissue (i.e., Barrett’s esophagus). Although nearly all EAC tumors are expected to arise in Barrett’s esophagus (BE) [4,5], only a small minority of EAC cases are diagnosed in patients actively enrolled in BE surveillance programs designed to catch cancers earlier [6]. Consequently, current BE surveillance strategies do not benefit most patients who eventually progress to EAC, and improvements in both early detection and effective monitoring are needed to provide this benefit.

One potential contributor hypothesized to contribute to the dramatic increase in both EAC and BE is a shift in the esophageal microbiome in Western populations. The subsequent role that this altered microbiome may have in promoting progression to EAC is far from defined. Here we review the current knowledge regarding evolution and progression of BE to EAC, with a focus on the status of microbiome research in BE and EAC, highlighting current challenges and providing future research directions. 

## 2. Epidemiology of Barrett’s Esophagus and EAC

The only established precursor to EAC is BE [7], and successive histological stages of low-grade dysplasia (LGD) and high-grade dysplasia (HGD) increase the risk of developing EAC within premalignant BE. Underlying BE is likely responsible for all EAC [4,5]; this implies that to prevent EAC and understand its early stages of progression, we need to first better understand the neoplastic transformation of BE. 

The estimated prevalence of BE in the general population is 1–2% [8,9]. The pathogenesis of BE involves the replacement of normal stratified squamous epithelium in the distal esophagus with specialized columnar tissue in response to repeated acid reflux exposure. Certain high-risk populations are at increased risk of BE. In patients with gastroesophageal reflux disease (GERD) symptoms, the prevalence of BE increases dramatically, estimated at 7.2% [10]. Similar to trends in EAC, BE prevalence increases with advancing age [11] and occurs 3:1 in males:females [12]. In the United States in 2006, non-Hispanic whites had the highest crude incidence rate of BE (39/100,000) followed by Hispanics (22/100,000), Asians (16/100,000) and blacks (6/100,000) [13]. Due to the nearly identical risk factors between BE and EAC, the rise in EAC cases is likely due in part to a simultaneous rise in BE cases, many of which are undiagnosed in the population since BE itself is asymptomatic [14]. In previous studies, BE incidence was found to be increasing linearly from 1996 to 2003, then became stable from 2003 to 2012 in the United Kingdom (UK) and the Netherlands [12]. In the US, there is evidence of a similar trend that would coincide with evidence for a stabilizing BE incidence in the last decade [2,11,13]. However, there are still gaps in knowledge regarding the quantification of incidence rates of BE in the US population, how these rates are potentially still changing, and how the recommended surveillance of BE impacts EAC risk. 

Only 7.3% of patients have been previously diagnosed with BE at their initial EAC diagnosis, indicating under-screening and imprecise identification of the true at-risk population [15]. Patients with a prior BE diagnosis have much better survival (HR for all-cause mortality 0.39, 95% CI 0.27–0.58 [15]) mainly due to tumors being caught at a lower stage; 19% of patients who had a prior BE diagnosis were diagnosed with late-stage (stage III or IV) EAC compared to 44.5% of patients without a prior BE diagnosis [15]. This implies that surveillance of patients who have a BE diagnosis has a positive impact, but additional underlying factors contributing to improved survival may not yet be fully defined. 

In order to properly survey patients with non-dysplastic BE for development of EAC, the standard of care includes an upper endoscopy with quality metrics every 3 years [16]. This practice has limitations yielding high costs to healthcare, risks posed to the patient, and the psychological impact associated with a precancer diagnosis (i.e., dysplasia) for patients without evidence of overt benefit with respect to reduced EAC incidence/mortality. Recent clinical trials assessing improved sensitivity of initial BE detection using the non-endoscopic method Cytosponge-trefoil factor 3 (TFF3) shows promise for implementing expanded screening efforts. The Cytosponge-TFF3 procedure consists of patients swallowing a gelatin capsule attached to a string with a 3 cm wide mesh sphere compressed inside, then the thread is pulled up through the mouth approximately 10 min later [17]. Fitzgerald and colleagues found an improved detection of BE in patients in the Cytosponge-TFF3 intervention group with 2% (140/6834) of patients receiving a BE diagnosis compared with <1% (13/6388) of patients receiving a BE diagnosis in the usual care group in an intention-to-treat analysis [17]. An easily obtained non-endoscopic method that may be useful in the diagnosis of BE and/or the prognosis for defining risk of progression to EAC is the collection of saliva samples to determine risk of BE and/or future neoplasia. In the following section, we describe results from one study that quantified microbial populations within saliva samples as a diagnostic biomarker of BE; this could potentially then be used as an initial screening tool for determining patients who would benefit from an upper endoscopy [18]. 

If patients with BE do progress and early stage EAC is diagnosed, endoscopic eradication treatment (EET) is recommended to be performed with curative intent, which entails endoscopic mucosal resection (EMR) or endoscopic submucosal dissection (ESD) of visible lesions/nodules within BE followed by ablative techniques such as radiofrequency ablation (RFA) or cryotherapy to eradicate the remaining BE [19,20]. Patients with cancer precursors such as dysplasia may also be candidates for recommending EET, where the intent is to prevent or delay the onset of EAC by eradicating the precursors including the BE segment itself [21]. Guidance with respect to appropriate patient selection for EET continues to evolve [16,20]. To this end, it warrants emphasis that less than 0.3% of patients diagnosed with BE progress to EAC annually in the US [22]. Therefore, most patients with BE will not benefit from surveillance, and the potential small benefit may be outweighed by patient risk, inconvenience, psychological impact, cost, and inefficient resource allocation associated with interval endoscopic surveillance. With accurate determination of which genetic, microbial, or environmental factors in patients with BE increase the likelihood of progression to EAC, clinical recommendations could be adapted to focus on patients at highest risk, decreasing exams in lower risk patients. Additionally, any distinct factor driving progression from BE to EAC could be targeted for a preventative treatment.

## 3. Potential Role of Esophageal Microbiome in BE and EAC

The dramatic increases in both EAC and BE may be partially caused by shifting trends in the relative abundances of particular bacteria comprising the esophageal microbiome, and the role that these modern microbiota may have in fostering progression to EAC in some patients. The esophageal microbiome makes up only a small part of the human microbiome, which altogether contains nearly 40 trillion microorganisms including bacteria, archaea and fungi in addition to viral genomes [23]. The vast majority of the human microbiome consists of bacteria located in the large intestine. Different microbes can thrive in slightly different microenvironments across the human body with varying oxygen and pH levels. In recent years, microbe-targeted therapies such as antibiotics, prebiotics, probiotics, and fecal microbiota transplantation (FMT) have shown some success in colorectal cancer and other diseases along the gastrointestinal tract. For example, probiotics have been shown to counteract oral and gastrointestinal inflammatory lesions caused by cancer therapeutics [24]. Patients taking a probiotic for 6 months post colorectal resection surgery had a significant reduction in pro-inflammatory proteins when compared to a control group (*p* < 0.05) [25]. FMT can be used to alter a patient’s microbiome and in some cases can shift the colonic microbiome back to a higher diversity ‘healthy’ state. In the case of *Clostridiodes difficile* infection, a severe type of diarrhea, FMT was able to shift the microbiome back to a normal state and prevented the reoccurrence of the disease in 86.8% of cases [26]. If microbes influence the pathogenesis of EAC or the development of BE, a microbially targeted therapy may similarly be effective in preventing EAC progression. Moreover, each person’s individualized and highly distinct microbial community may affect pathogenesis, which would support the development of personalized microbe-targeted therapeutics in the future to prevent or treat EAC. From an evolutionary perspective, microbial populations in the oral cavity likely affect abundances within the esophageal microbiome due to migration between the two niches. The potential for contamination also necessitates caution when sampling from both anatomical locations. For example, this topic has been assessed in the context of the lung microbiome when distinguishing microbiota from bronchoalveolar lavage fluid and contemporaneous oral wash [27]. In a recent randomized controlled trial, the oral microbiome and esophageal microbiome were shown to be tightly linked as assessed by oral swabs and esophageal brushings that were sterilized at the beginning of the upper endoscopy [28]. This study found that intervention with chlorhexidine mouth rinse over 2 weeks caused significant alterations in the oral microbiome when compared to the untreated group (Wilcoxon rank-sum *p* = 0.013 for pairwise distances measured with weighted UniFrac), and this intervention also changed the expression of several genes associated with inflammation measured by RNAseq of esophageal tissue [28]. No study has yet fully revealed whether changes in the microbiome could relieve gastroesophageal reflux symptoms, or if BE metaplasia creates a different microenvironment that attracts different types of microbes compared to the normal esophagus [29]; thus, further investigation is warranted. 

Some changes in the microbiome may also be linked to demographic risk factors for EAC. A previous study has proposed that changes in the microbiome co-occurring with obesity increase inflammation in the gastrointestinal (GI) tract [30], potentially providing an ideal environment for the development of EAC that may explain at least in part why obesity is a risk factor for EAC [31]. Additionally, certain ratios of common upper GI bacteria have also been associated with waist-to-hip ratio [32], a risk factor that is more strongly associated with increased risk of BE onset than Body Mass Index [33]. Furthermore, if certain microbial communities are unique to BE, there may be an opportunity to develop a microbe-specific biomarker to identify more patients with BE in the population. In a study comprised of 49 patients (17 controls, 32 patients with BE), saliva samples were assessed with 16S rRNA gene amplicon sequencing (16S), and a logit model including three genera of bacteria distinguished samples from patients with BE from controls with an area under the receiver operating curve (AUROC) of 0.94 (95% CI 0.85–1.00) [18]. Larger studies are needed to test whether assessment of the oral microbiome using saliva samples could be used as a reliable screening tool for BE.

### 3.1. Barrett’s Esophagus

**Figure 1:**
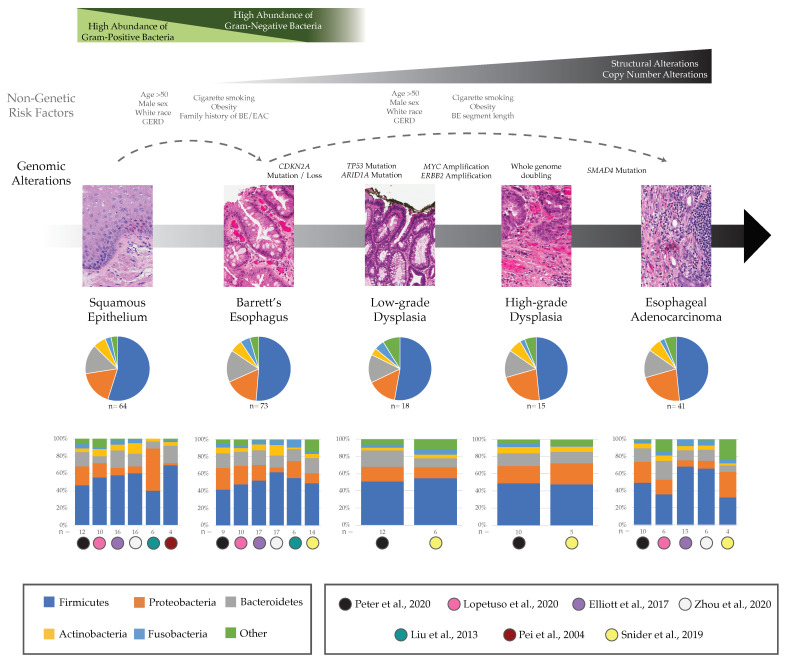
Microbial and genomic changes in the progression from BE to EAC. The stages of progression from normal tissue to EAC with corresponding non-genetic risk factors for BE and BE-EAC progression [7,40,41,42,43], common genomic changes frequently detected [44,45,46,47,48,49,50,51] as well as general trends expected in increasing Gram-negative bacterial species in BE [38,52]. Relative abundances reported across the phylum level at each stage are provided in aggregate (pie charts) and in each study individually (bar plots) to highlight study-specific heterogeneity [34,35,36,37,38,39,53]. Note, technical differences including analysis pipelines can lead to differences among studies beyond the biological differences likely to be present in the samples. Additional methods would need to be applied to distinguish methodological differences from cohort or stage differences across studies. Recent analysis methods such as differential ranking can help resolve stage differences and identify clinically significant microbial changes [54]. Hematoxylin and eosin stain images courtesy of Matthew Stachler, UCSF. GERD, gastroesophageal reflux disease; BE, Barrett’s esophagus; EAC, esophageal adenocarcinoma.

Previous research has explored characterization of microbial communities within patients with BE, and we will focus on six studies to highlight the main findings in the field. These studies used 16S, a low-cost sequencing technology commonly used for extracting abundance, diversity, and phylogenetic information from microorganisms within a sample. Although 16S is frequently used in microbiome studies, it does have the limitation that it only reflects the relative abundance of microbes in the sampled area, and does not indicate the mechanistic or functional role that each taxon plays in the health of the sampled tissue. Three of the six studies used biopsy samples obtained during endoscopic exams [34,35,36], one study used esophageal brushings [37], one used both biopsy and brushings [38], and one study used both techniques along with samples obtained using the Cytosponge device [39]. Relative abundances reported across the top five most abundant microbial communities at the phylum level from normal to BE patients in these six studies are displayed in Figure 1. 

At the genus level, a study of Japanese patients found that the most abundant bacteria in non-BE esophageal tissue was the genus *Streptococcus* of the Firmicutes phylum, a Gram-positive species that had a relative abundance of 21% in patients without BE or reflux esophagitis (*n* = 6 controls) compared to a lower 11% relative abundance in BE samples (*n* = 6) [36]; this decreasing trend was confirmed in two other BE studies [35,39]. According to the same study of Japanese patients, the most abundant bacterial genus in patients with BE was a Gram-negative bacteria, *Veillonella* of the Firmicutes phylum, which had relative abundance of 19% in patients with BE but was not found in normal patients [36]. Although group differences in this small study were not statistically significant [36], the same two trends were supported using culture-independent methods by Lopetuso and colleagues considering biopsies from 10 control patients versus 10 patients with BE [35]. In contrast, a study from the UK did not find an increase in proportional abundance of *Veillonella* in tissue biopsies from patients with BE versus control patients with reflux [39]. In terms of alpha diversity, the UK study showed there was a modest decrease in diversity measured by the Shannon index in samples taken with the Cytosponge device from patients with BE compared with control samples (*p* < 0.05) [39], while other studies found no statistically significant differences in diversity levels comparing BE with control samples [34,35,37].

One recent study confirmed an earlier finding by Yang and colleagues that patients with BE have significantly higher amounts of Gram-negative bacteria compared to control patients with normal esophagus [38,52] although this has not been consistently replicated in other studies [37,39]. Finally, Blackett and colleagues found that biofilms from patients with BE (*n* = 45) had an increased relative abundance of *Campylobacter* genus of the Proteobacteria phylum compared with those from patients with non-BE gastroesophageal reflux disease (GERD) (*n* = 37; *p* = 0.0814) [55]. Using culture-independent methods, an increase of an unclassified species of *Campylobacter* in BE patients versus controls was also seen in bacteria from brushings analyzed by Snider et al. [37] but the opposite trend in relative abundance of this genus was found in fresh frozen tissue samples in the UK study [39]. This genus is often found in the mouth but rarely in the normal esophagus and there is still debate surrounding the ability of *Campylobacter* to also cause DNA damaging nitrosative and oxidative stress [55].

### 3.2. Esophageal Adenocarcinoma

There has been a limited number of studies quantifying esophageal microbial communities in patients with EAC; we highlight five here [34,35,37,38,39]. All of these studies also considered BE samples, as referenced above. Relative abundances at the phylum level across the progression from BE to EAC, including two studies that additionally considered stages of LGD and HGD, are further highlighted in Figure 1. In a comprehensive study across all stages of BE-EAC progression, Snider and colleagues analyzed esophageal brushing samples from control patients (*n* = 16), patients with BE (*n* = 14), LGD (*n* = 6), HGD (*n* = 5) and EAC (*n* = 4) and found that the most drastic shift in the microbiome occurred between stages of LGD and HGD [37]. Together, patients with HGD or EAC had similar relative abundances of microbial communities with a significant increase in the Proteobacteria phylum (32.1 vs. 17.7%, *p* = 0.04) and decrease in the Firmicutes phylum (38.3 vs. 55%, *p* = 0.04) when compared to the group of patients with BE or LGD [37]. Specifically, there was an increase in the family Enterobacteriaceae of the Proteobacteria phylum in the HGD/EAC group compared with the BE/LGD group (*p* = 0.02) [37]. The increase in Enterobacteriaceae is of interest because it has been linked with inducing inflammation in the gut of patients with inflammatory bowel disease (IBD) and may be having a similar effect in the esophagus of patients with EAC [37,56]. Finally, Snider et al. found the species *A. muciniphila* of the Verrucomicrobia phylum to be present only in the HGD/EAC group (22% of samples) and it was not found in the BE/LGD group. Notably, this species has been associated with increased tissue inflammation that may contribute to the progression to EAC, but has also shown beneficial effects in protecting mice from diet-induced obesity [37,57].

In pairwise comparisons, Snider et al. found that patients with EAC have decreased microbial alpha-diversity using the Simpson index compared to patients with BE (*p* = 0.006) and even those with LGD (*p* = 0.01) and HGD (*p* = 0.01) [37]. Concordant with this, Elliott and colleagues found decreased alpha-diversity in patients with EAC compared with BE and controls using the Shannon index (Kruskal-Wallis *p* = 0.0075) [39]. When evaluating beta-diversity, the authors showed that EAC phylogenies cluster away from those of the control samples using the Bray-Curtis measure (*p* = 0.002, parsimony test) reflecting that there was a significant difference between microbial populations across EAC and control samples [39]. Lopetuso et al. also showed significant differences in the Bray-Curtis measure between both EAC and controls (*p* = 0.018) and between EAC and BE (*p* = 0.034) [35], while two remaining studies did not find significantly distinct clusters using beta-diversity calculated across all groups [34] nor between the BE/LGD group versus HGD/EAC group [37]. Overall, these initial findings support the potential role of the microbiome in the evolution from normal to BE to EAC but studies, particularly for EAC, are still limited in number, sample size, and ability to establish causality. 

### 3.3. Proton Pump Inhibitors

Some studies have specifically considered patients actively taking proton pump inhibitors (PPI) and the potential effects these may have on the esophageal microbiome. PPIs are a common pharmacotherapy to relieve symptoms of GERD, heal esophagitis or peptic ulcer disease (PUD), eradicate *Helicobacter pylori (H. pylori)*, as well as serve as prophylaxis to prevent PUD in high-risk patients. PPIs are often prescribed for short-term use, but there are few indications for long-term use. PPI use has been associated with reduced risk of BE progression, therefore most patients with BE are prescribed PPIs. For all patients actively taking PPIs (which includes BE, LGD, HGD, EAC and controls), one study found a reduced relative abundance of Gram-negative bacteria (51.1 vs. 67.3%, *p* = 0.05) and increased relative abundance of *Streptococcus* (*p* = 0.03) when compared with patients not taking PPIs (*n* = 10 control patients) [37]. Thus, patients actively taking PPIs may have microbiomes that are similar to those of patients without BE. Gram-negative bacteria are often associated with promoting chronic inflammation, so the decrease in Gram-negative bacteria by using PPIs may be helping to prevent EAC but the exact preventative role of these medications is debated. Notably, many population studies, including the American Gut Project [58], have seen PPIs among the pharmaceuticals that are associated with greatest change in the gut microbiome.

### 3.4. Helicobacter pylori

Another open question in the microbiome field concerns the presence and consequence of *H. pylori* on the esophageal microenvironment versus its known pathogenic role on the stomach microenvironment. *H. pylori* is a Gram-negative bacteria within the Proteobacteria phylum and thrives in an acidic environment with relatively low oxygen levels between 2 and 5% [59]. *H. pylori* is considered a class I carcinogen by the World Health Organization due to its established causal link in noncardia gastric cancer [59,60]. Because it is also causally associated with peptic ulcer disease (PUD), international societies recommend eradication when active *H. pylori* infection is diagnosed. *H. pylori* has high genetic diversity though most studies have focused on VacA, CagA, and BabA strains. Certain strain-specific constituents have been associated with greater virulence and carcinogenicity. Specifically, compared to *H. pylori* CagA negative strains, the CagA-positive strains have been associated with a higher risk of noncardia gastric cancer, and may also be the strains that confer protection against EAC [61]. It is important here to note that although *H. pylori* is positively associated with risk of gastroduodenal PUD and noncardia gastric cancer, [59] it is inversely associated with risk of adenocarcinoma of the gastric cardia, a cancer with nearly identical risk factors to EAC. Interestingly, rates of cardia gastric cancer are also increasing in countries such as Korea and Japan where rates of noncardia gastric cancer are declining in the face of mass *H. pylori* eradication campaigns [62]. Indeed, this rise in cardia gastric cancer may also be related to incorporation of elements of a Westernized diet, increasing rates of obesity and metabolic syndrome, among other factors.

Some studies report that the 5-fold increase in EAC cases in the United States over the past four decades might be due at least in part to the dramatic decrease in *H. pylori* across the population with the widespread use of antibiotics [1,14,63]. The decrease in *H. pylori* is reflected as a cohort effect in the US with each new generation having less *H. pylori* over time; 30.8% of patients born 1930–1939 had *H. pylori* infection compared with 18.6% of patients born in 1960–1969 [64]. One study by Anandasabapathy and colleagues found the absence of *H. pylori* was associated with risk of advanced stages of HGD and EAC within BE (*p* = 0.06) [65]. Another study by Islami and colleagues found the CagA-positive strain of *H. pylori* to be inversely associated with EAC risk (OR 0.41 95% CI 0.28–0.62) [61]. Blackett et al. reported that patients with EAC had a statistically significant lower abundance of *H. pylori* compared with normal control, and patients with GERD or BE [55], implying that the reduction in *H. pylori* may be permissive of cancer transformation. Interestingly, one study that considered both *H. pylori* status and DNA content measured by flow cytometry found that patients with BE who were *H. pylori* negative had a statistically significant higher rate of aneuploidy versus patients with BE who were *H. pylori* positive (*p* < 0.045) [32]. High rates of aneuploidy are predictive of progression to EAC over time in this same patient cohort [44,45], suggesting a potential role for *H. pylori* in the negative selection of cells that stochastically accrue chromosomal alterations. In gastric cancer development, *H. pylori* infection may also influence inflammatory response through DNA methylation of gene promoters [66,67] and enhance expression of beta-defensins that play a role in host response and the co-evolution of precancerous lesions within the gastric microbiome [66,68]. Finally, when *H. pylori* is absent in the gastric microbiome of patients, the gastric microbiome reflects the microbiome of patients with BE more closely than patients without BE due to the increased amounts of Gram-negative bacteria, such as those in the Proteobacteria phylum, and decreased amounts of bacteria in the Firmicutes phylum [14]. Overall, the debate regarding the causal role of *H. pylori* in the development of BE and subsequent EAC persists and more mechanistic studies are needed in this area. 

## 4. Current Challenges

We discuss a set of fundamental questions below regarding microbial influence in BE-to-EAC development but note that many of these challenges are also generalizable more broadly to other premalignancies of the gastrointestinal tract.

### 4.1. How Do Microbial Communities Influence Precancer and Cancer Genomes, Specifically during the Early Growth of EAC Malignancies?

The genomes of BE cells are known to become highly altered during their evolution [46], with many somatic mutations (e.g., *TP53* inactivation) [47,48], chromosomal alterations and rearrangements [44,45], and epigenetic modifications [49,50] (e.g., age-related hypermethylation) accruing during the progression from BE to EAC (Figure 1). If microbes are influencing both BE and EAC pathogenesis, we need to understand the mechanistic role in potentially driving the selection of cancer-primed (epi)genotypes during this dynamic process of somatic evolution. Random mutations accrue due to DNA replication errors but the potential influence of microbes on the Darwinian selection of certain mutated clones is uncertain because joint analyses of host and microbial genomes in the same samples are currently lacking. Do inter- and/or intra-cellular microbes increase mutagenesis, or can they be used as spatial markers for where mutations are most likely to reside in cells within a tissue? Recent studies have considered the utility of the microbiome in cancer diagnostics [69,70]; however, there is still a significant gap in addressing the potential for cancer prediction in patients with premalignant conditions, such as patients with BE who have not yet developed EAC. Although cancers are initiated in single cells via genetic and epigenetic changes that produce a malignant phenotype, Poore et al. showed a difference between cancerous and healthy tissue strictly using the corresponding microbial DNA in 32 cancer types within The Cancer Genome Atlas (TCGA), including esophageal carcinoma and colorectal adenocarcinoma, with area under the receiver operator characteristic curves (AUROC) > 0.80 in 32 different cancer types [69]. Further, the authors found that tissue-specific microbiome classifiers using machine learning algorithms applied to whole genome sequencing (WGS) data can discriminate between cancer stages I and IV in colon adenocarcinoma (AUROC = 0.80, Area Under the Precision Recall curve [AUPR] = 0.81), stomach adenocarcinoma (AUROC = 0.86, AUPR = 0.75), and kidney renal clear cell carcinoma (AUROC = 0.88, AUPR = 0.74). Notably, the colon is often used for microbiome research because samples are easy to collect noninvasively and it has at least two orders of magnitude more bacterial content compared to all other organs [71]. A challenge remains to determine the utility of microbiome diagnostics for other cancer types where the microbial communities are less abundant, such as esophageal communities that have 10,000 bacteria per mm^2^ compared with the colon that has nearly 100 billion bacteria per mL [53,71].

Another important question to consider is whether the microbiome itself is causing cancer progression or if these microbial populations simply thrive in the environment of the cancer tissue after it has transformed. Our current understanding is incomplete regarding how microbes thrive on or inside cancer tumor cells, and if they may be promoting tumor spread. A recent study in mice by Bertocchi and colleagues found that certain microbes within a lung tumor microenvironment went on to colonize a new site in the liver and then encouraged the subsequent recruitment of metastatic cells to the new site [72]. Before we can conclude that microbes expedite cancer growth or cause metastases, however, more experimental models are required to disentangle differences between the association versus causation of microbes in cancer. Most studies thus far considering patients with EAC and their accompanying microbiome were association studies, where no active changes to the microbiome were attempted in study subjects. Often these studies are performed by collecting biopsies, esophageal samples or oral swabs from patients during routine endoscopy examinations, then sorting patients into groups based upon symptoms or diagnosis and analyzing the microbial associations across groupings. Ideally, more controlled studies focusing on causative mechanisms are needed, such as the one performed by Münch and colleagues. These authors used a mouse model of BE and confirmed that a high fat diet promoted tumors by inducing inflammation and shifting the esophageal microbiome [73]. Replicating this in studies using human trials will be particularly difficult due to the challenges of collecting frequent microbial samples longitudinally from the esophagus and ensuring they are not contaminated with oral saliva if those populations are diluting the signal specificity (see Table 1). Overall, a better combined understanding of the microbial populations in the EAC microenvironment and within EAC cells, along with the co-occurring complex genomic profiles of human DNA within EAC tumors, is needed. These studies will need to consider both the microbial and host genetics within EAC patients. 

### 4.2. What Evolutionary Forces Shape Microbial Communities throughout the Body, and Specifically in BE and EAC?

It is well established that microbial communities live on and within all organisms on Earth, are associated with a wide variation of both beneficial and harmful effects, and undergo Darwinian evolution and natural selection [79,80]. However, we have an incomplete understanding of how particular bacteria species survive, evolve, and can thrive in certain abundances within the human body. Prior studies of the gut microbiome demonstrate that the host environment and self-selection across microbial communities lead to stable balanced communities within the human gut [81]. Infants form a relatively stable community around 31 months post-birth [82]. Studies using model organism systems, such as murine and compost, found evidence from analysis of mathematical models of evolution that changes in microbiota communities present within different environments can be explained by processes of neutral evolution [83]. These changes in population structure can be modeled using equations derived from stochastic processes with a set number of mechanistic parameters that can also include the strength of selection pressures [84]. Previous studies have found similar evidence for neutral evolution in the microbiomes of the urological tract [85], skin [86], and healthy lung tissue [87]. It is still an open question whether the acidic environment that BE engenders in the lower part of the esophagus allows for a selective advantage of certain microbial species over others, and mathematical models of microbial evolution will aid in our future understanding of this complex process.

**Figure 2:**
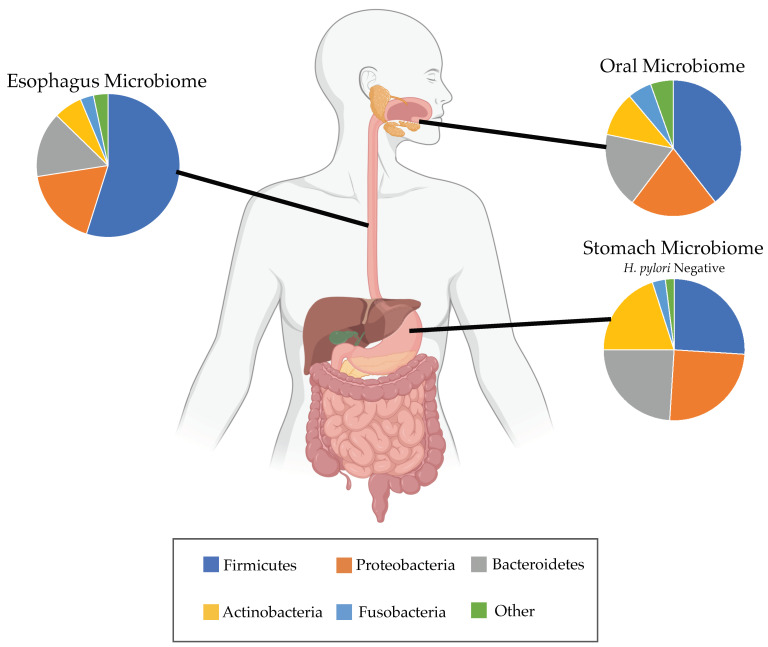
Microbiome of a healthy patient. Microbial abundances at the phylum level from patients without Barrett’s esophagus or cancer shown for the oral [14,18,89], esophageal [34,35,36,38,39,53] and *H. pylori* negative gastric [14] microbiomes. Esophageal microbes can migrate from and between the stomach and oral cavity, therefore influencing the expected diversity of the esophageal microbiome in clinical studies.

In many ways, the evolution of microbial populations is more complex compared to host cell evolution due to the fact that new microbial species can migrate into the body and likely to different locations in the body more easily. The constant movement of microbes throughout the body leads to fluctuation in the local diversity of microbial species, which can further reflect the health of the host. As mentioned previously, Elliott and colleagues found a decrease in microbial diversity comparing EAC tissue to normal reflux or dyspepsia samples (*p* = 0.0075) [39], and this decrease may be a sign of a small number of species dominating the microbiome. In the gut, decreased diversity is often sign of an unhealthy gut microbiome that has been shown to increase chances of obesity and type 2 diabetes [88]. The microbial communities living in the healthy esophagus are physically surrounded by somewhat distinct abundances of microbes in the microenvironments present in the stomach and oral cavity (Figure 2). Understanding how certain microbes migrate to the distal esophagus will serve as an important aspect to consider if aiming to change the course of microbial evolution and affect the residing populations. Overall, more longitudinal studies in humans are needed to track temporal changes of microbial populations in order to gain necessary insight on how abundances evolve toward certain population equilibria, where they migrate in the body, and why some organs are preferentially colonized over others.

### 4.3. How Can We Increase the Number of Longitudinal Clinical Datasets That Capture Evolutionary Dynamic Changes in the Esophageal Microbiome?

Collecting esophageal samples in diverse patient cohorts over longitudinal timepoints can be a clinically demanding task due to technical challenges (see Table 1). Additionally, when using microbial data in studies there are other challenging factors such as host contamination, variation in microbial samples from AM to PM hours, and discrepancy caused by sampling during fasting and postprandial periods. One way to increase the number of datasets available for esophageal microbiome research would be to implement easier methods for collecting samples. Currently, patients enrolled in studies undergo sampling in the esophagus with endoscopy brushings or forceps biopsies [90]. Both of these methods are invasive for the patient, and thus collecting microbial samples in a more efficient and less costly manner is a present challenge for data generation and analysis. The Cytosponge device has successfully measured the majority of genera found in biopsies and brushings, sampling a larger surface area but also capturing microbes from the proximal stomach and oral cavity [39]. Saliva and oral swabs also provide another sampling technique for potentially detecting microbial changes in BE or EAC. It is important to note that there is a much larger abundance of microbes in the oral cavity compared to the esophagus so saliva would likely represent mainly oral microbiota rather than esophageal-specific microbial communities [90]. In a randomized controlled trial, bacteria from oral swabs have been shown to be highly correlated with the esophageal microbiome within the same patient but this association was weaker when using saliva samples [28]. Wang and colleagues showed that the oral microbial taxa differed when comparing saliva samples from healthy patients versus those with reflux esophagitis, making it a potential biomarker for a diagnosis of reflux esophagitis [91]. If there is similar potential for biomarkers and diagnosis in the saliva of BE [18] and EAC patients, saliva samples would be a much simpler way to collect data and expand databases of consequential microbiota. Although additional research is needed to determine whether saliva samples could confirm a BE or EAC diagnosis, saliva samples and oral swabs proffer a potential avenue for more microbial datasets to be created in patients who do and do not progress to EAC. 

## 5. Future Directions

To address the challenges above, we highlight the following important research efforts that will shape microbial research areas in BE and EAC patients in the future. By creating open access datasets and improving the accuracy of microbiota classifications that can be detected with metagenomic assays, more researchers will be able to incorporate microbiome characterizations into future research allowing us to uncover the relationships between microbes and causes of EAC. 

### 5.1. Open Access to Large Microbial Datasets Integrated with Host Genomics

Publicly available microbial data is currently accessible on various platforms including the NCBI database, the European Nucleotide Archive, and Qiita, an open-source microbial study management platform that allows researchers to analyze and share their data [92]. One example dataset within these databases is the American Gut Project, which involved the collection of fecal, oral and/or skin samples along with a detailed questionnaire about dietary and lifestyle habits from over 10,000 citizen scientists in 57 countries [58]. The American Gut Project, along with the majority of publicly available microbial data, was created from 16S experiments. Whole genome sequencing (WGS) data can also be used to address microbiome research questions while, in some cases, simultaneously enabling the characterization of host genomes. Although the American Gut Project has been extraordinarily successful and is still leading to scientific breakthroughs, because the main dataset consists of 16S and because IRB approval covers only microbial analysis, host associated genomics does not accompany the microbiome data [58], and this remains true for the WGS data currently being obtained in the American Gut Project and the new international umbrella project that encompasses it, The Microsetta Initiative. In contrast, the goal of the NIH Integrative Human Microbiome Project (iHMP) was to better understand the human microbiome by creating longitudinal datasets from both microbiomes and host cell genomes, and to integrate functional data from metagenomics and metatranscriptomics [93]. Studies within iHMP focused on three main health issues: preterm birth, the onset of IBD, and the onset of type 2 diabetes. Although iHMP did not focus on cancer patients, it can be used as an exemplar for future host and microbial studies in cancer and precancer. In particular, metabolomics data in addition to microbial datasets from stages of progression from BE to EAC would provide functional insight because metabolic products of particular species are likely mediators for stage-specific changes in phenotypes [94]. Notably, panels of serum metabolites incorporated into multivariate models have previously achieved moderate discrimination between patients with HGD or EAC versus patients with BE without HGD or EAC (AUROC = 0.75) [95].

As the microbiome field continues to grow, more industrial partners and government grants are providing the appropriate resources to help accelerate research findings [96] and collect more microbial and host samples (for example, by establishing collaborative microbiome centers [97]). With more samples available to analyze across increasing numbers of locations in the human body, it will be possible to disentangle both the evolution of microbes from the environment and between different anatomical niches, and to consider their influence in tandem with host genomics. To address the role of microbial evolution in the progression from normal to BE to dysplasia to EAC in particular, future studies will require longitudinal samples in progressors and non-progressors.

### 5.2. Improve Detection of Microbes at the Species Level

The vast majority of publications considering the esophageal microbiome used 16S to analyze the microbial species in study samples. Although this is often sufficient in sites such as the colon where there are large concentrations of bacteria, it may be less effective when considering sites such as the esophagus that has a much lower concentration of bacteria. One solution is to use technologies such as whole genome shotgun sequencing (WGS). WGS can be used to sequence microbial communities by using host-removal library preparation methods allowing for the enrichment of the non-human reads with downstream analysis focusing solely on bacterial DNA. However, this method is considerably more expensive compared with 16S sequencing, especially when performed with much greater sequencing coverage (tens of millions of sequences per sample, rather than thousands). In a study comparing WGS and 16S, WGS yielded a higher number of genera observed (mean = 948.75, 95% CI 913.04–986.46) compared with 16S (mean = 224.5, 95% CI 187.63–263.37) in soil samples [98]. Additionally, samples analyzed using WGS had a significantly higher alpha diversity on average compared with 16S [98]. Although shotgun WGS at low depths can still detect differences among alpha and beta diversities between samples [99,100,101], repurposing WGS from human cancer studies for detection of diverse microbial DNA using bioinformatics pipelines is possible in moderate-to-high depth samples [69] but similar results using available samples at low depths (~1x) have not yet been demonstrated (Table 1). Finally, defining the human microbiome within precancer and cancers is still a growing field and computational tools for analysis, including machine learning methods for classification and diagnostics, hold great potential for clinical impact but are still in development along with best practices for cleaning and analyzing microbial data. Currently this gap in suitable metrics adds complexity to comparing results across studies because there may be drastic variation across processed datasets, along with often limited information provided in publications and repositories regarding the quality of the datasets analyzed [102].

## 6. Conclusions

There is clear potential and value for the expansion of microbiome research in the cancer field, specifically in EAC and its precursors where there have been less studies performed overall compared to studies on the microbiome of colorectal cancer. Three example scenarios where this expansion would be beneficial are: (1) quantifying risk of BE development in patients with GERD based on microbial populations; (2) determining if it is possible to risk stratify patients with BE who are at high-risk versus low-risk of developing future EAC based on microbial profiles so that adjustments to surveillance and interventions can be made; and (3) testing our ability to intervene and shift the microbial communities in the esophagus to improve the outcome of some patients with EAC. Based on current findings that cancer-specific microbial populations are detectable in cancer patients [69], this is a promising area of early detection capability, particularly in those with curable precursors such as detected dysplasia in BE patients. Carcinogenesis is a complex process of clonal evolution with many factors influencing cell population trajectories, including changes in cell genotypes that lead to advantageous phenotypes, and changes in the immune infiltrate and stromal factors that contribute to a cancer-promoting microenvironment. Efforts to understand microbial changes within cancerous and precancerous cells, and within their surrounding microenvironment, will help solve another piece of this puzzle.

## Figures and Tables

**Table 1:** Benefits and pitfalls of esophageal microbiome sampling methods and most common microbial sequencing methods. References provided for each example.

Benefits		Pitfalls	Publications
**Sampling Methods**			
**Esophageal Biopsies**	Considered the ‘gold’ standard Less possibilities for cross-contamination between oral microbes Ability to sequence the host cells in addition to microbes	Invasive and poses increased risk (if performed outside of standard of care endoscopy) Expensive for the hospital and patient Low abundance genera can be difficult to detect	[37,39]
**Esophageal Brushings**	Found to have improved quality and quantity of microbes (e.g., number of OTUs) detected compared to biopsy, potentially due to enrichment of bacteria on epithelial surface Larger surface area samples can be taken from patient compared with biopsies	Invasive and poses increased risk (if performed outside of standard of care endoscopy) Expensive for the hospital and patient Further validation required before being used often in practice Only detects microbes on the surface level	[32,37]
**Saliva/Oral swabs**	Minimally invasive Low cost Evidence that BE patients have a distinct oral microbiome	Detects the oral microbiome and not specifically esophagus populations More large-scale studies are needed to confirm the accuracy of using saliva samples	[18]
**Cytosponge samples**	Samples large surface area Patients in clinical trials overall have reported acceptability of the procedure Detects majority of genera detected with brushings and biopsies Minimally invasive Can be taken in a doctor’s office in 5–7 min	May not detect as many microbes compared to other methods Requires validation in larger studies before being clinically available Decreased esophageal specificity: samples the esophagus, but likely also proximal stomach and oral cavity	[39,74,75]
**Sequencing Methods**			
**16S rRNA Sequencing**	Largest number of tools and pipelines to analyze data Well-established databases Relatively inexpensive to run Remains accurate with high levels of host DNA within a sample	Only identifies bacteria and archaea Taxonomic resolution is often limited to the genus or family level, not species Amplification bias from polymerase chain reaction (PCR)	[76,77,78]
**Whole Genome Shotgun Sequencing**	Identifies bacteria, fungi and virus all in one run Richer taxonomy compared to 16S High confidence in species and strain identifications Provides functional profiling	More complex bioinformatic methods Fewer databases available Risk of host cell contamination More expensive than 16S Increased chance of false positives	[76,77,78]
**Whole Genome Sequencing (Human DNA library prep)**	Large number of public datasets available for which microbes have not yet been explored Corresponding studies often quantify genomic alterations in the same sample Enables joint analysis of microbial population abundances and human DNA mutations	Low microbial yield—other than in fecal and gut samples, removal of over 90% of data required due to non-microbial reads and contamination Often no control for microbial contamination was performed in original study Lack of control samples for microbiome specific studies	[69]

## Data Availability

Data sharing not applicable.
